# Injection of high dose botulinum-toxin A leads to impaired skeletal muscle function and damage of the fibrilar and non-fibrilar structures

**DOI:** 10.1038/s41598-017-14997-3

**Published:** 2017-11-07

**Authors:** Jessica Pingel, Mikkel Schou Nielsen, Torsten Lauridsen, Kristian Rix, Martin Bech, Tine Alkjaer, Ida Torp Andersen, Jens Bo Nielsen, R. Feidenhansl

**Affiliations:** 1 Center for Neuroscience, University of Copenhagen, Copenhagen, Denmark; 2 Niels Bohr Institute, University of Copenhagen, Copenhagen, Denmark; 3 Medical Radiation Physics, Clinical Sciences, Lund University, Lund, Sweden; 4 Present Address: European XFEL, Hamburg, Germany

## Abstract

Botulinum-toxin A (BoNT/A) is used for a wide range of conditions. Intramuscular administration of BoNT/A inhibits the release of acetylcholine at the neuromuscular junction from presynaptic motor neurons causing muscle-paralysis. The aim of the present study was to investigate the effect of high dose intramuscular BoNT/A injections (6 UI = 60 pg) on muscle tissue. The gait pattern of the rats was significantly affected 3 weeks after BoNT/A injection. The ankle joint rotated externally, the rats became flat footed, and the stride length decreased after BoNT/A injection. Additionally, there was clear evidence of microstructural changes on the tissue level by as evidenced by 3D imaging of the muscles by Synchrotron Radiation X-ray Tomographic Microscopy (SRXTM). Both the fibrillar and the non-fibrillar tissues were affected. The volume fraction of fibrillary tissue was reduced significantly and the non-fibrillar tissue increased. This was accompanied by a loss of the linear structure of the muscle tissue. Furthermore, gene expression analysis showed a significant upregulation of COL1A1, MMP-2, TGF-b1, IL-6, MHCIIA and MHCIIx in the BoNT/A injected leg, while MHVIIB was significantly downregulated. In conclusion: The present study reveals that high dose intramuscular BoNT/A injections cause microstructural damage of the muscle tissue, which contributes to impaired gait.

## Introduction

Botulinum toxin (BoNT/A) is among the most potent toxins to humans that are known^1,2^. One gram of crystalline preparation of BoNT/A can potentially kill 1.000.000 people. In several incidents its use as a biological weapon has been attempted^3^. Nevertheless, BoNT/A is the first biological toxin that has been licensed for treatment of human disease after the pioneering work of the ophthalmologist Alan Scott who used BoNT/A for the treatment of strabismus^4^. Today, the list of diseases that are treated by using BoNT/A is long and includes cervical dystonia^5^, blepharospasm^6^, urinary incontinence^7^, anal fissure^8^ and numerous movement disorders^9^, including cerebral palsy^10^, migrane^11^, depression^12^, mandibular recontouring^13^ and, fascial wrinkles^14^. Furthermore, BoNT/A continues to be the most common minimally invasive procedure performed by plastic surgeons and has during the past decade grown into a billion dollar industry^15^.

BoNT/A injection is also commonly used against the development of contractures in patients with central motor lesions. However, only a few studies have investigated the effect of BoNT/A injections on the muscle at tissue level. Skeletal muscle consists of the contractile proteins myosin and actin, which are incorporated into thick and thin filaments, respectively. Together they form arrays in longitudinally repeated banding patterns termed sarcomeres. Sarcomeres in series form myofibrils, and many parallel myofibrils exist in each fiber. A muscle contraction occurs when an action potential reaches the presynaptic terminal of a motor neuron. This activates voltage-dependent calcium channels and allows calcium ions to enter the neuron. Calcium ions bind to proteins (synaptotagmin) on synaptic vesicles, triggering vesicle fusion with the cell membrane and subsequent neurotransmitter release from the motor neuron into the synaptic cleft. The motor neurons then release acetylcholine (ACh), which diffuses across the synaptic cleft and binds to nicotinic acetylcholine receptors (nAChRs) on the cell membrane of the muscle fiber, also known as the sarcolemma. The binding of ACh to the receptor depolarizes the muscle fiber, and triggers a series of molecular events that includes the binding of calcium to the muscle-regulatory proteins, and causes the interaction between myosin and actin filaments, and subsequently the formation of cross-bridges causing muscle contraction.

Botulinum toxin works by blocking the release of acetylcholine from presynaptic motor neurons, and this chemical denervation causes a cascade of downstream events in the muscle thus causing muscle paralysis^16^. BoNT/A asserts its effect by proteolysis of the SNARE protein synaptosyme-associated protein of 25 kDa (SNAP25) in the synapses of the motor neurons^17^. SNAP25 is a cytoplasmic protein, which is crucial in the fusion between the membranes of the vesicles containing ACh and the cell membrane at the axon terminal. When BoNT/A is injected into muscle tissue, the proteolysis of SNAP25 prevents the exocytosis of ACh, and effectively leads to muscle paralysis^2,18,19^.

**Figure 1:**
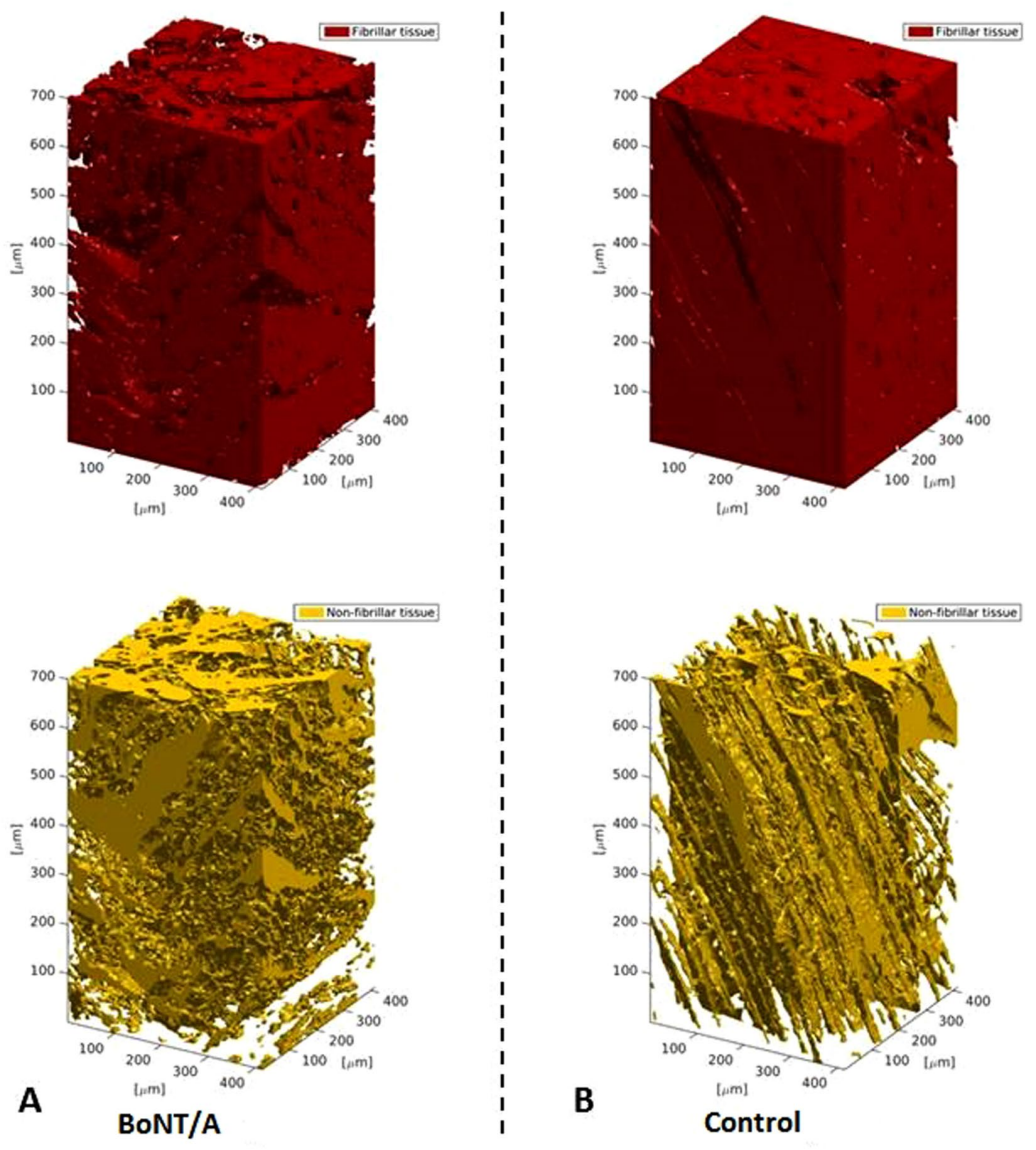
3D visualizations of tomograms from the SRXTM measurements. Shown are the fibrillar (red) and non-fibrillar tissue (yellow) of the muscle in BoNT/A injected (**A**) and control leg (**B**) of one of the rats (3 weeks post injection of 6UI BoNT/A (3 × 20 pg BoNT (2UI)/a ∼ 60 pg BoNT/A in total). The SRXTM data indicate that BoNT/A injection causes damage to the muscle tissue structure and organization.

Muscle atrophy has been noted as a common side effect as a result of denervation^20,21^. Furthermore, considerable fiber atrophy has been observed after BoNT/A (5–10 UI) injections into the longissimus dorsi muscle of rabbits^22^. One rat study investigated changes in different muscle proteins after BoNT/A injections (dosage of 5 units/kg body weight) and observed that the expression of various proteins changed after injection when compared to saline injections^23^. In fact, thirty-eight proteins were associated with alterations of energy metabolism, contractile function of the muscle, transcription and translation, cell proliferation and cellular stress response^23^. In addition, intramuscular BoNT/A injections (Each dose was 6 U/kg, in a 100 µL volume) have been shown to induce significant changes of fiber type composition with a shift from faster to slower isoforms^24^. Nonetheless, it is still unclear whether or not the fibrillar structure of the muscle fibers is damaged as a result of BoNT/A injections. To resolve this issue a 3D visualizations of the tissue is required. Standard lab-based X-ray tomography does not have the sufficient X-ray brilliance to resolve the details of tissue at microstructural level within a reasonable time frame. However, Synchrotron Radiation X-ray Tomographic Microscopy (SRXTM) dramatically improves the sensitivity within biomedical applications^25,26^. The tunable-energy monochromatic beam allows us to achieve high sensitivity to small variations in mass densities. This makes SRXTM well-suited for imaging biological tissue in contrast to conventional X-ray CT^25^. Within medical science, the modality has been successfully applied to study the microstructure of a range of tissues such as the 3D brain anatomy in murine model^27^, the microvascular network and thrombi in hepatocellular carcinoma^28^ and hyaline cartilage in human knees^29^. Hence, the SRXTM is ideal for the purpose to monitor tissue changes in muscles after high dose BoNT/A injections. In this study, x-ray attenuation and phase contrast was taken into consideration by implementation of the Paganin approach^30^. The aim of the present study was to explore the effects of high dose intramuscular BoNT/A injections on healthy muscle tissue using SRXTM. This was done to confirm whether or not the muscle tissue maintained its integrity and microstructure following BoNT/A injections. In Fig. 1 an example of a 3D reconstruction of the fibrillar and the non-fibrallar tissue is shown for a BoNT/A injected muscle and the corresponding control. The scale is 400 µm × 400 µm × 700 µm. It is clearly seen that the BoNT/A injection causes significant changes in the microstructure of the tissue. Most obvious is that the ratio between fibrillary and non-fibrillar tissue changes and that the linear structure of the muscle tissue is lost. In order to quantify these changes we calculate the volume fraction of the fibrillary tissue and the isotropy index as defined below. As secondary study aims we also investigated the effects of high dose BoNT/A injections on both the gait pattern and the gene expression profile in healthy rats.

## Results

### Volume fraction

**Figure 2:**
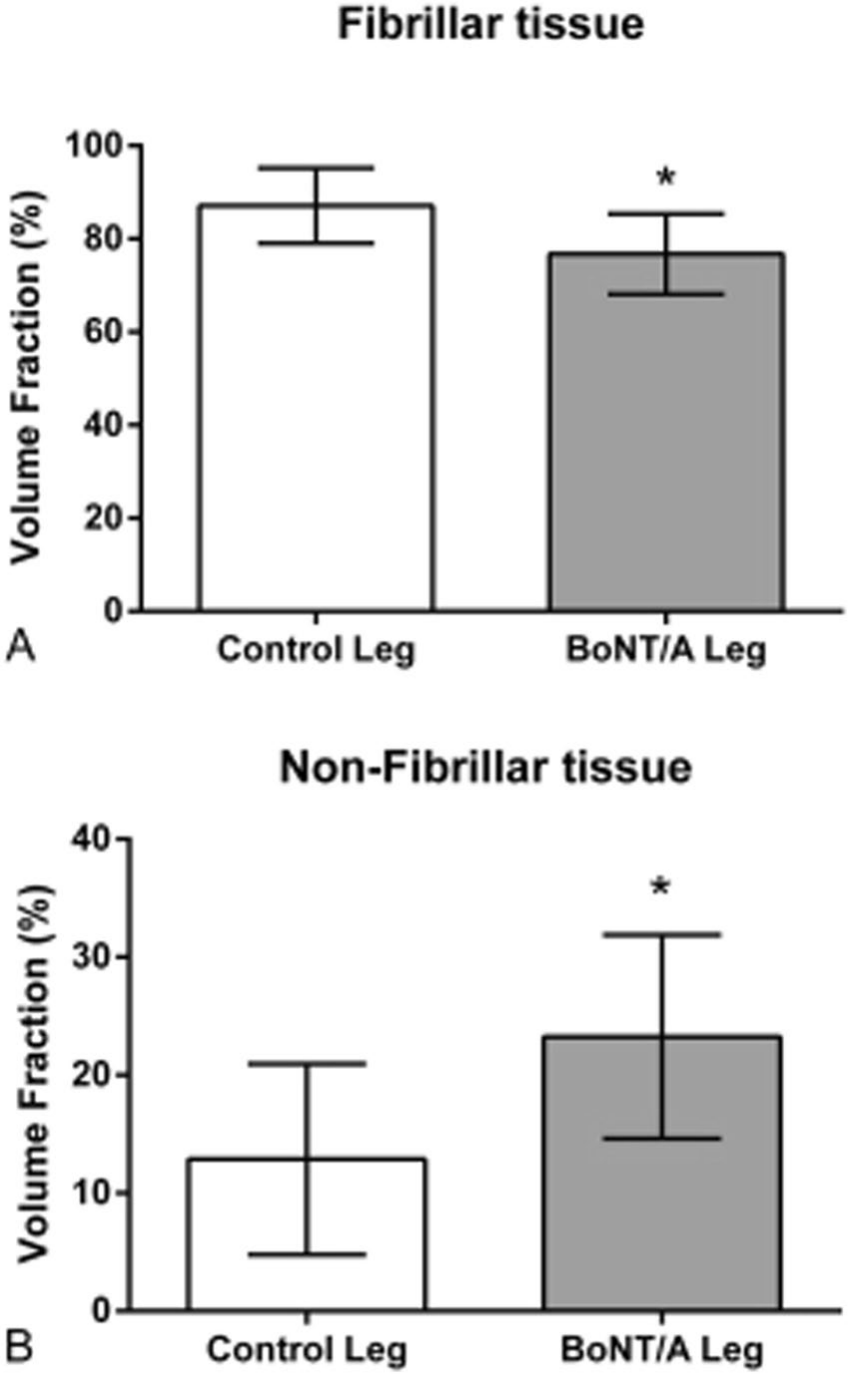
Volume fraction in percent of the fibrillar (**A**) and non-fibrillar tissue (**B**) of the muscle. All data are shown as Mean ± SEM. *Indicates a significant difference between BoNT/A and Con leg. Dose of injection 6UI BoNT/A (3 × 20 pg BoNT (2UI)/a ∼60 pg BoNT/A in total). The level of significance was p < 0.05.

The muscle tissue organization was severely affected by the BoNT/A injections 3 weeks after injection (Figs 1 and 2) of 20 pg BoNT/A per muscle head (60 pg in total). The amount of the fibrillar tissue significantly decreased (p = 0.02), while the non-fibrillar tissue was significantly increased (p = 0.02).

### Muscle atrophy

**Figure 3:**
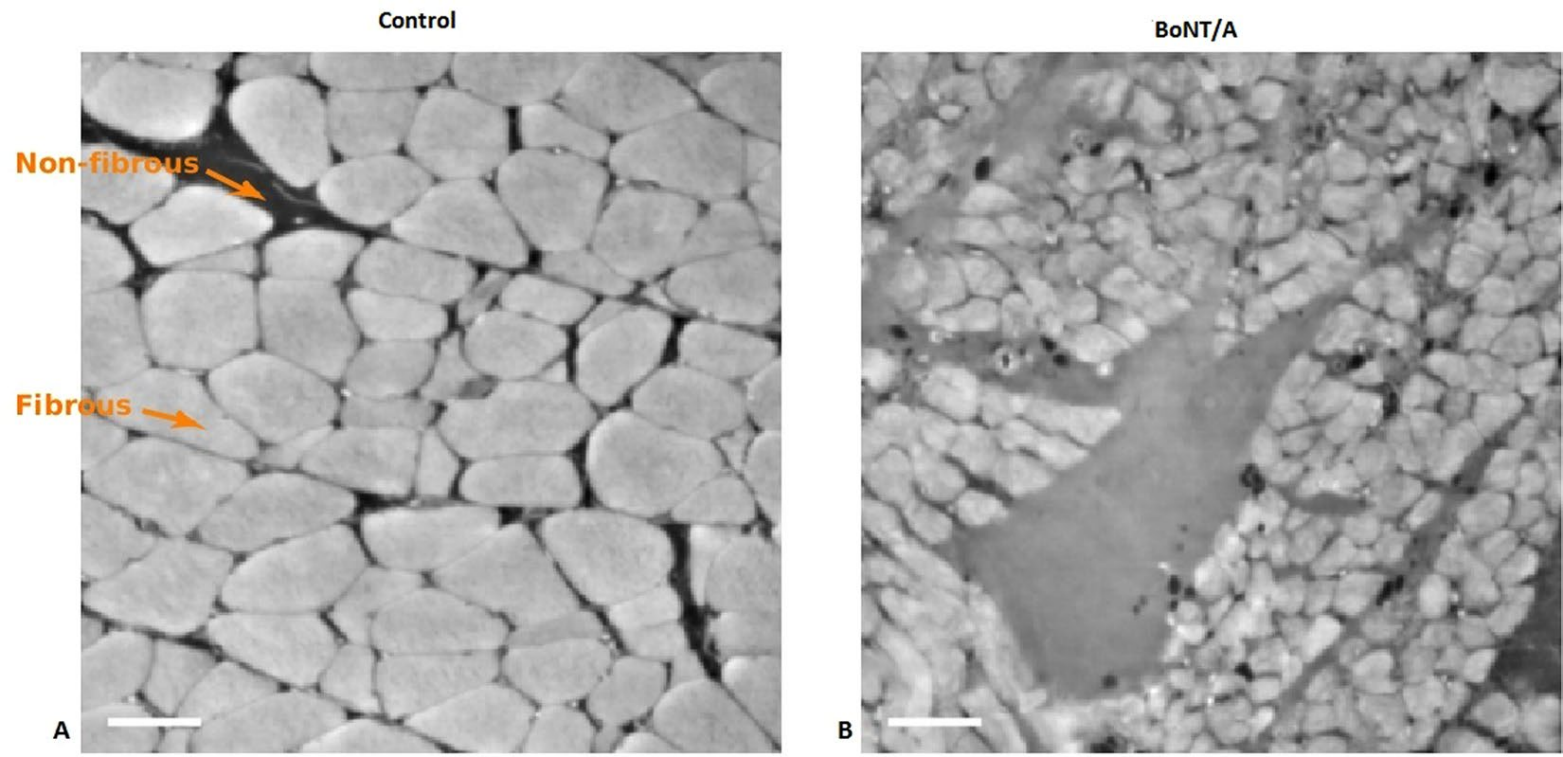
2D images of muscle cross sections. Left image control leg, right image BoNT/A injected muscle (3 weeks post injection). The BoNT/A injected muscle contains smaller muscle fibers than the control muscle, indicating muscle atrophy. Arrows are indicating fibrous and non-fibrous tissue. Dose of injection 6UI BoNT/A (3 × 20 pg BoNT (2UI)/a ∼60 pg BoNT/A in total). The scale bar equals 50 µm.

Three weeks after BoNT/A injection the wet weight of the triceps surae was decreased significantly (p = 0.00005) by 45% (BoNT/A injected leg (BoNT/A): 0.78 ± 0.03 g and Contralateral saline injected leg (Con): 1.43 ± 0.07 g) in the BoNT/A injected leg. A 2D image of the muscle crossections displays the atrophy observed in the BoNT/A leg (Fig. 3).

### Anisotropy

**Figure 4:**
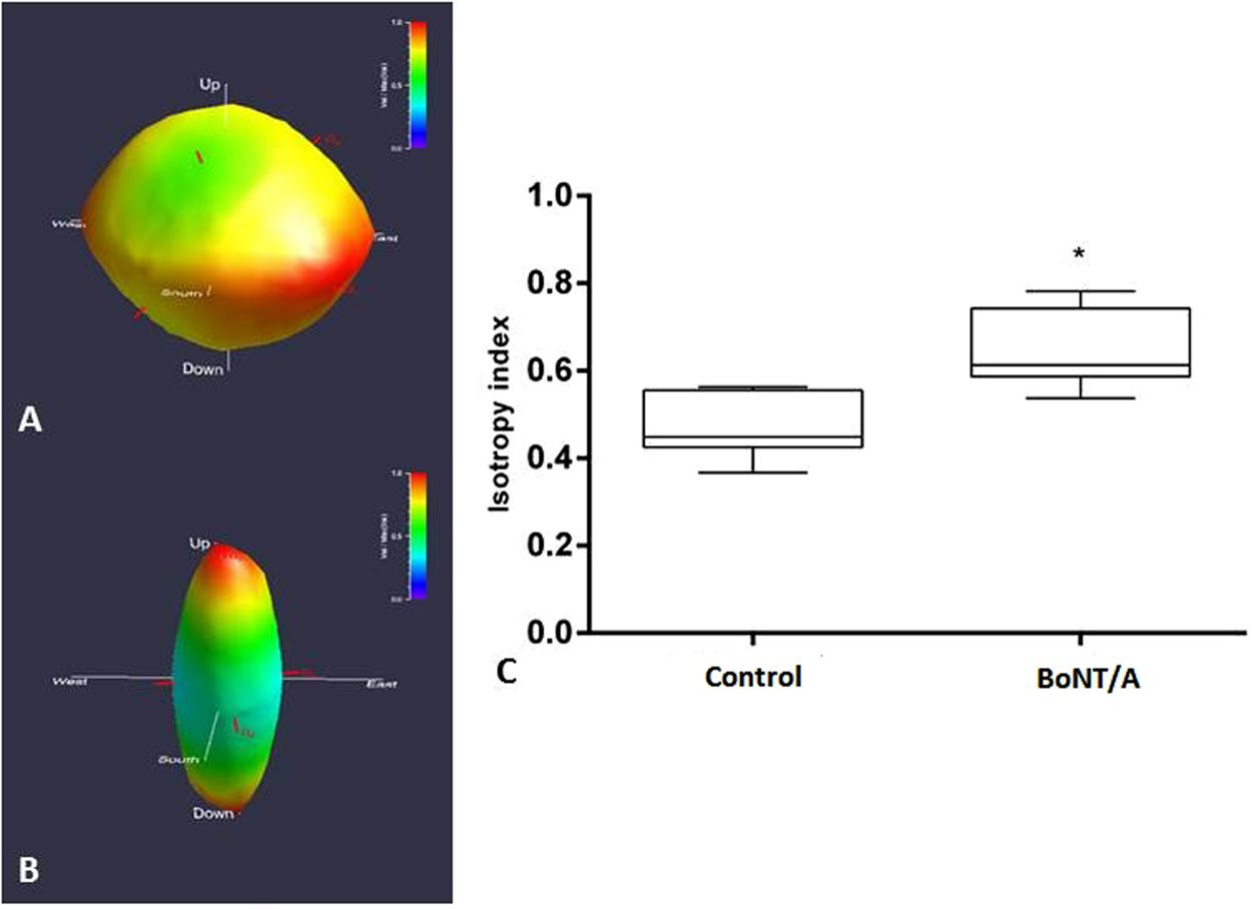
Star length distribution (SLD) rose diagrams depicting non-fibrillar tissue orientation in BoNT/A (**A**) and control (**B**) muscle from one of the rats (3 weeks post injection). Dose of injection 6UI BoNT/A (3 × 20 pg BoNT (2UI)/a ∼60 pg BoNT/A in total). Distance from origin and colour (violet = minimum, red = maximum) indicate relative component value. Red axes show principal component directions and relative magnitudes. The boxplot (right **C**) shows the isotropy index of n = 6 rats shown as Mean ± SEM. *Indicates a significant difference between BoNT/A and Con leg, showing an significantly increased anisotropy index after the BoNT/A injections, which indicates a loss of linear structure of the muscle tissue. The level of significance was p < 0.05.

The isotropy index from the star length distribution (SLD) analysis significantly increased (p = 0.002) after the BoNT/A injections (3 weeks post injection), which indicates a loss of linear structure of the muscle tissue. This was further supported by visualization of the 3D orientation of the muscle tissue using rose diagrams (Fig. 4). The diagram displays the strength of directions of the non-fibrillar tissue in the volume of interest (VOI). For linearly structured tissues, the diagram will take the shape of a strongly elongated ellipsoid whereas isotropic tissues with uniformly distributed directions will take the shape of a sphere. As seen from Fig. 4, the BoNT/A injection has resulted in a loss of directionality of the non-fibrillar tissue.

### Gait pattern analysis

**Figure 5:**
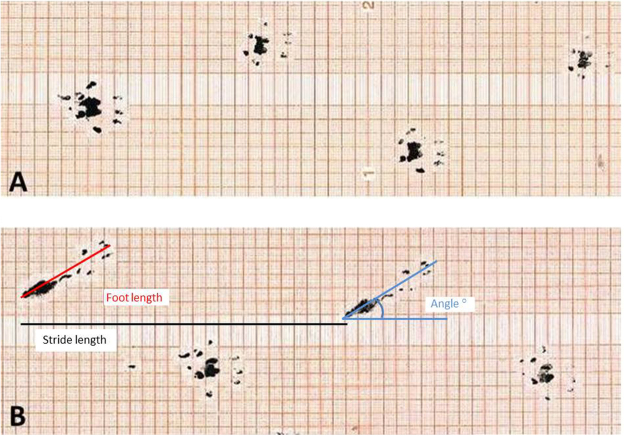
Foot step analysis. The hind paws were dipped in ink and the rats ran over graph paper at (**A**) baseline (2 days pre injection), and (**B**) post (21 days) after BoNT/A injection into the left triceps surae. Dose of injection 6UI BoNT/A (3 × 20 pg BoNT (2UI)/a ∼60 pg BoNT/A in total). The strife length, foot angle and the foot length were analyzed. All parameters were significantly affected in the BoNT/A leg when compared to baseline and the control leg (p < 0.05).

**Figure 6:**
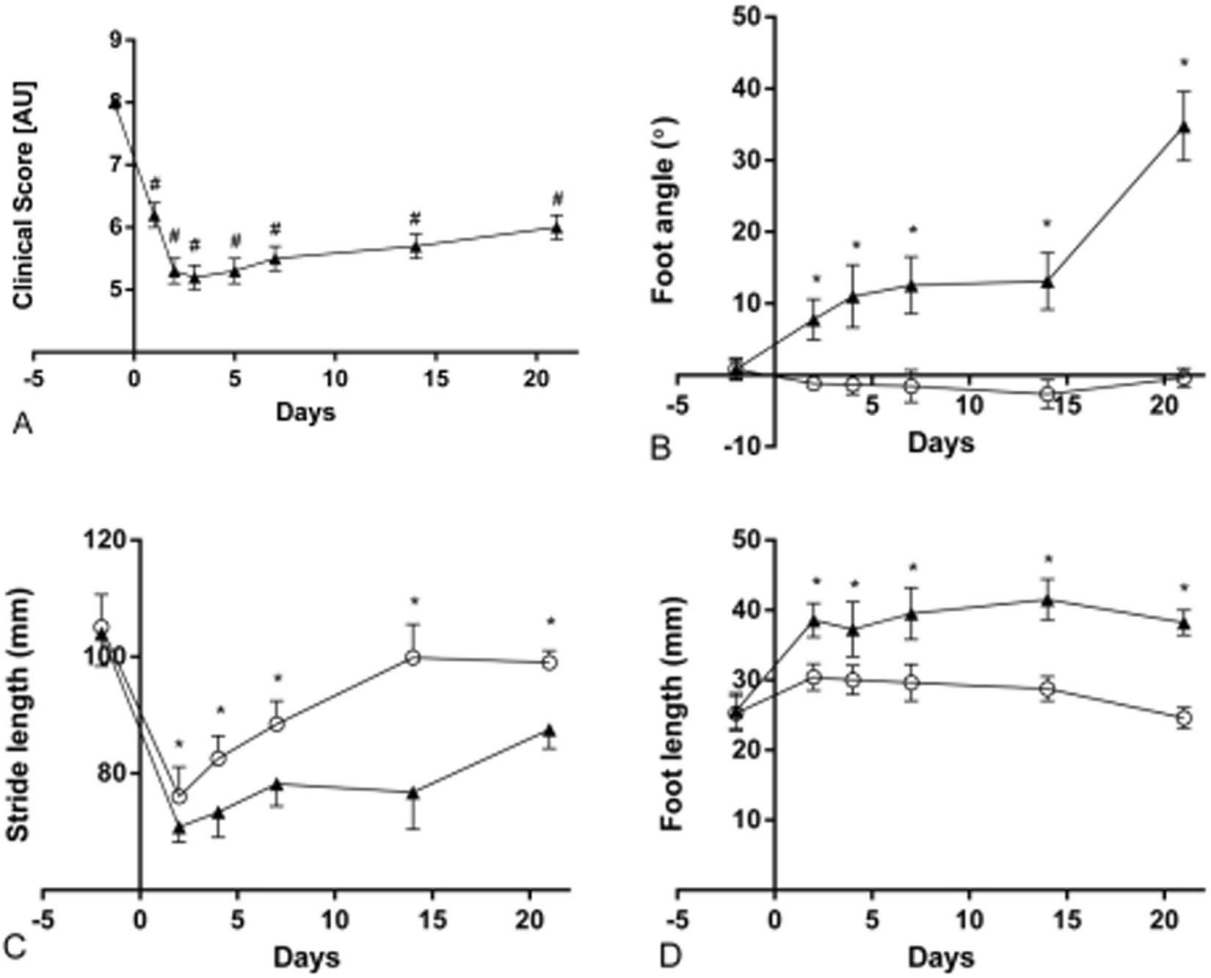
The stride length, foot angle and foot length was analyzed as indicated on Fig. 5. The clinical score (**A**) shows all animals in arbitrary units AU. The foot angle (**B**) is shown in degrees (°). The stride length (**C**) is shown in mm. And the foot length is shown in mm. In both B,C and D the black triangles represent the BoNT/A injected leg, and the open circles represent the contralateral Con injected leg. Dose of injection 6UI BoNT/A (3 × 20 pg BoNT (2UI)/a ∼60 pg BoNT/A in total). All data are shown as Mean ± SEM. *Indicates a significant difference between BoNT/A and Con leg, ^#^indicates a significant difference from baseline. The level of significance was p < 0.05.

The gait pattern of the rats was significantly affected. There was a significant main effect of both time (p < 0.0001) and leg (p = 0.0008) on the stride length but not of the interaction. Across time, the stride length was significantly reduced for the BoNT/A leg compared to the non BoNT/A leg (p = 0.0006). Across legs, the second pre-test stride length value was significantly higher than any post-test values (p < 0.0006 for all comparisons). No significant differences were observed between the post-tests. There was a significant main effect of time, leg and the time-leg interaction on the foot length (p < 0.0001 in all cases) indicating that the rats became flat footed after BoNT/A injections. The post hoc test revealed that for the BoNT/A leg the foot length was significantly increased in all post-test observations when compared to the pre-test observation (p < 0.0001 in all cases). For the non BoNT/A leg, the foot length was significantly shorter at the pre-test compared to the first three post-tests (p = 0.004, p = 0.012 and p = 0.028, respectively). However, the last post-test foot length did not differ significantly from the pre-test. The foot length of the BoNT/A leg was significantly greater at all post-tests compared to the non BoNT/A leg (p < 0.0001 in all cases). The foot angle was measured as indicated in Fig. 5. A positive foot angle indicates external rotation, and a negative foot angle indicates an internal rotation of the foot. There was a significant main effect of time, leg and the time-leg interaction on the foot angle (p < 0.0001 in all cases). The post hoc test revealed that for the BoNT/A leg all post-test observations were significantly larger than the pre-test observation (p < 0.002 in all cases). For the non BoNT/A leg, the foot angle did not significantly change from the pre-test to the post-tests. The foot angle of the BoNT/A leg was significantly greater at all post-tests compared to the non BoNT/A leg (Fig. 6).

### Clinical score assessment

The clinical score dropped significantly already 24 h post injection (p = 0.0001) and reached the lowest level on day 3 post injection (p = 0.00001). The rats never recovered during the protocol and the clinical score was still decreased three weeks post injection. There was a significant main effect of time on the clinical score (p < 0.0001). The post hoc test revealed that all post-test observations were significantly lower compared to the pre-test observation (p < 0.0001 in all cases) (Fig. 6).

### Gene expression

**Figure 7:**
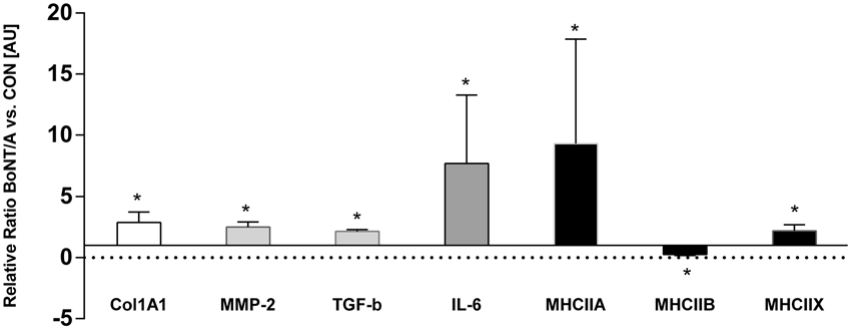
The relative changes of gene expression after botulinum toxin injection. The control leg equals 1 and all expressions are shown relative to the expression of the control leg. All data are shown as Geo Mean ± SEM. *Indicates a significant difference between BoNT/A and Con leg. Dose of injection 6UI BoNT/A (3 × 20 pg BoNT (2UI)/a ∼60 pg BoNT/A in total). The level of significance was p < 0.05.

The gene expression of seventeen gene targets were analyzed in n = 8 rats (Fig. 7). Specific gene targets were selected covering the areas of muscle structure, muscle metabolism, Extracellular matrix components, connective tissue breakdown and muscle fiber types. There was a significant main effect of both BoNT/A treatment (p < 0.001) and gene (p < 0.001) on the gene expressions and a significant interaction (p < 0.001). Post hoc tests revealed a significant upregulation of gene expression in: collagen type 1 (*COL1A1*), interleukin 6 (*IL-6*), transforming growth factor beta 1 (*TGF-β1*), matrix metallopeptidase 2 (*MMP-2*), myosin heavy chain IIA (*MHCIIA*) and myosin heavy chain IIX (*MHCIIX*). Myosin heavy chain IIb (*MHCIIB*) was significantly downregulated in the BoNT/A leg compared with the control leg.

### Body weight

**Figure 8:**
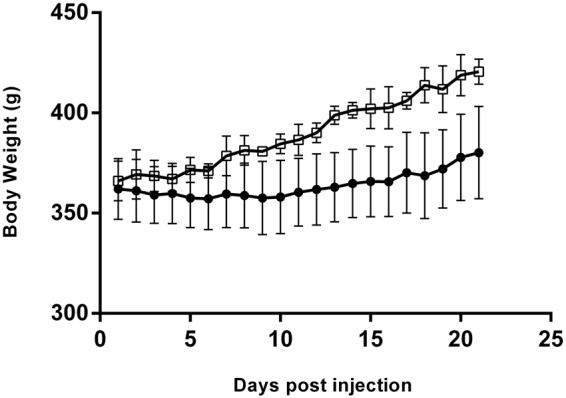
The development of the body weight of the rats after botulinum toxin injection. The open squares represent control rats (n = 2), and the black dots represent BoNT/A rats (n = 6). All data are shown as Mean ± SEM. *Indicates a significant difference between BoNT/A and Con leg. The level of significance was p < 0.05.

The development of the body weight differed significantly between the BoNT/A injected animals and the Control group (p < 0.0001) (Fig. 8). In addition both groups changed significantly over time (after 1 week) (p < 0.0001).

## Discussion

The main finding of the present study is that the microstructure of the skeletal muscle showed signs of muscle damage following high dose BoNT/A injections (3 × 20 pg BoNT (2UI)/a ∼60 pg BoNT/A in total (6UI)), both at the fibrillar- and non fibrillar level of the tissue. The 3D tomographs showed that the tissue lost its linear structure, and that the structural composition was clearly affected. The anisotropy analysis of the tomograms gives further support to these indications. The isotropy index increased after high dose BoNT/A injection, which indicates that the muscle tissue was more randomly oriented following high dose BoNT/A injections.

Botox is used in several different disciplines with many different purposes. In addition, it is becoming more and more common for healthy individuals to receive BoNT/A injections, not to cure a disease, but in order to improve the appearance. BoNT/A is among others used to sculpt different muscles of the body, including the masseter muscles for mandibular recontouring (used for when individuals complaint of a “squared” lower face^31^ or the medial and lateral gastrochnemius for calf recontouring (for when individuals complaint of prominent calf muscles)^31^. However, recent studies have expressed some concerns regarding the use of BoNT/A^32–34^.

The chemical denervation induced by BoNT/A injection prevents muscle contraction and causes a cascade of downstream events in the muscle e.g. inflammation, satellite cell activation, oxidative stress, atrophy and metal ion imbalance^35,36^. Whether BoNT/A also has a direct effect on the muscle tissue is unclear. However, it has been shown that repeated BoNT/A injections (once a day for 28 days at doses of 1, 3, and 9 ng kg-1 day-1) result in pronounced muscle atrophy^37^ and muscle weakness. In the present study we observed a loss of 45% muscle wet weight after three weeks, and we have previously shown that muscle force drops significantly after BoNT/A injection using the same dose as in the present study^33^. In fact several studies report muscle weakness as the most common treatment-related adverse event^31^. Furthermore it has been shown that side effects are temporary and often related to usage of high doses^38^. Even though the use of BoNT/A has been reported to be safe and many patients are treated without complications, some studies report unwanted side effects^39,40^. One study by Blaszczyk *et al*. examined unwanted side effects (adverse events AEs) after BoNT/A injections in 79 cerebral palsy patients^41^. Altogether 95 AE’s were reported in 45 patients. Nineteen patients reported muscle weakness, and four patients did report severe adverse events including speech disorders, swallowing difficulties and respiratory troubles. These findings indicate that side effects after BoNT/A injection are quite common^41^. Cerebral palsy patients are often treated with BoNT/A in order to improve gait function, and several studies have shown that this treatment is safe and without side effects^10,42^. However, we suggest that the success of the treatment should be evaluated with care. We propose that even though patients do not report any unwanted side effects, this does not mean that the BoNT/A treatment was successful in these patients per se. In the present study we observed that BoNT/A injections had a significant effect on the gait pattern of the present rats. The rats developed a flatfoot on the BoNT/A injected leg (Fig. 5). Furthermore the clinical score dropped already after 24 hours and did not return to baseline within three weeks (Fig. 6). The same observation has been made previously by Ozawa *et al*.^43^ after BoNT/A injection (dose 5 units/mL). In addition, the BoNT/A injection caused an external rotation of the foot possibly to regain balance (Fig. 6). Furthermore, it appears that more and more people question whether the few positive effects achieved by BoNT/A injection are actually overcome by the negative effects it has on the muscles^32,33,44^.

The affected gait after 24 hours is a result of the reduced presynaptic acetylcholine release that follows Botulinum toxin injection. BoNT/A cleaves a SNARE protein SNAP25, which is required for exocytosis and as a result several downstream events are affected including muscle atrophy. The muscle atrophy induces weakness, and the weakness affects the gait pattern. Thus while the gait pattern and clinical score at the early time points reflect the neural effects of BoNT/A, atrophy and muscle weakness is likely to contribute to the impairment of gait function at the later time points (3 weeks post injection).

Eamus *et al*. (1999) have shown that a single injection of BoNT/A into the calf increased the length of gastrocnemius during gait^45^. Furthermore, gait analysis of ninety-seven CP patients revealed that muscle strength was highly related to muscle function and explained more of the gait variance than spasticity^46^. Based on the present findings we suggest that the desired outcome of BoNT/A treatment in cerebral palsy patients should be reconsidered, and future studies should focus on finding a better treatment instead of improving a drug that does not entail the desired effects.

Furthermore, whether muscle contouring of the calf muscles influences the gait pattern in humans has not yet been investigated, but based on the present findings we suggest that it might be an important point to focus on in future investigations as well.

The damage that occurs in the muscle after high dose BoNT/A injection requires a comprehensive remodeling process. This is reflected in gene expressions of the present study (Fig. 7). Both the collagen synthesis and collagen breakdown is upregulated after BoNT/A injection. This finding is in agreement with previous observations showing increased collagen synthesis at gene expression level three weeks after muscle injury in rats^47^. On the other hand, immobilization causes a decrease of collagen expression^48^. Thus, the increased collagen synthesis might indicate an accelerated tissue remodeling after muscle damage rather than being a result of the denervation due to BoNT/A injection (Fig. 7). The anisotropy findings of the present study indicate a loss of tissue orientation, which further indicates that the microstructure of the muscle is undergoing an extensive remodeling process. Furthermore, the inflammation marker IL-6 was upregulated in the present study. IL-6 is a pleiotropic cytokine involved in tissue regeneration and remodeling, indicating an inflammatory response after BoNT/A injection in the muscle^49^. A significantly up-regulation of IL-6 following muscle injury, coinciding with the active period of muscle regeneration has been observed previously in mice^48^. The present upregulation of IL-6 in the BoNT/A injected muscle underlines that the high dose BoNT/A injection caused muscle damage. Furthermore, one study observed a significant increase of satellite cells after intramuscular botox injections in rabbits^50^. In resting muscle satellite cells remain quiescent, while muscle injury and trauma invokes activation of satellite cells^51,52^. Numerous studies have shown that skeletal muscle satellite cells are essential for muscle fiber repair and regeneration^53–55^. Muscle wasting occurs in a variety of conditions, including muscular dystrophies, cancer cachexia and sarcopenia^56^. In the present study the intramuscular BoNT/A injection caused a decrease of muscle mass of (45%). In addition, muscle atrophy can affect specific fiber types, involving predominantly slow type 1 or fast type 2 muscle fibers (slow-to-fast or fast-to-slow fiber type shift)^57^. Age related muscle wasting (sarcopenia) induces a fast-to-slow fibertype shift^58,59^. Spinal cord injury patients on the other hand experience a type 1 fiber atrophy with a slow-to-fast fiber type shift due to disuse^60^. In the present study a significant upregulation of myosin heavy chain IIA and myosin heavy chain IIx expression was demonstrated, while MHCI was unchanged and MHCIIb was downregulated (Fig. 7). These results indicate that BoNT/A injections cause a slow-to-fast fibertype shift due to upregulation of fast fiber type expression rather than due to atrophy of type 1 fibers. This might be a compensatory process in order to compensate for a significant loss of muscle force and mass after BoNT/A injections which has been observed previously after a single injection of 100 μL of BoNT/A of 6.0 units/kg^61^. However, the present findings are in contrast to previous findings showing that BoNT/A injection in the gastrocnemius muscle of rats caused a fast-to-slow fibertype shift when rats received either 3,6,12 or 18 UI of BoNT/A^18^. These contradictory findings may be explained by the difference in how long after the BoNT/A injection the tissue was analyzed. The present findings reflect the early regeneration phase (3 weeks after injury), while the study by Dodd *et al*. demonstrates the long term effect of the damage after BoNT/A injection^18^. Furthermore, the present study investigated mRNA levels only. To clarify whether BoNT/A injections cause a fibertype shift both short- and longterm should rather be investigated using proper fibertyping methods, than mRNA expression only. In addition Fig. 3 shows qualitative transectional images of the microstructure of the muscle, and makes the effect of BoNT/A visible by eye showing that the muscle fibers in the BoNT/A injected leg are smaller and appear with blurred edges, indicating muscle atrophy of all present muscle fibers. By using Synchrotron Radiation X-ray Tomographic Microscopy (SRXTM) the present study revealed that the microstructure of skeletal muscle tissue was significantly damaged three weeks after a single injection of BoNT/A in rats. However, whether repeated injections would lead to even further damage needs further investigations.

Whether repeated injections of BoNT/A might have unwanted and irreversible effects is still unclear for several different treatments. There are to date unfortunately only few studies that have examined the effects on the muscle tissue after repeated injections. However, Minamoto *et al*. investigated the effect of repeated BoNT/A injections into the tibialis anterior muscle in rats and observed that a single injection of BoNT/A caused a 50% decrease of muscle torque, while a second injection of botox decreased the muscle torque to 95% when compared to the pre injection level^61^. The authors concluded that a second BoNT/A injection caused a profound and persistent loss in muscle function and altered muscle structure^61^. Unwanted side effects have also been reported in cosmetic medicine where cumulative and repeated injections into the masseter muscle for lower face contouring can cause different adverse effects including difficulty chewing, speech disturbances and muscle fatigue^31^.

In addition, the loss of bodyweight after BoNT/A injections might also be characterized as an unwanted side effect. In the present study, when the body weight of the rats that were injected with BoNT/A was compared with the bodyweight of healthy untreated control rats, it was observed that the body weight was significantly lower in the BoNT/A rats one week after the botox injection until the end of the protocol (Fig. 8). This indicates that a high dose of BoNT/A affects the bodyweight development of the rats, which might reflect an inhibited growth of the rats. Whether this is only experienced after injection of a high dose BoNT/A is unknown, but it is possible that a high dose of BoNT/A injection causes systemic effects which are reflected by the body weight of the rats. However, there is increasing evidence that BoNT/A inhibits growth both in rats and in humans^32,62,63^. Gough *et al*. has previously claimed that BoNT/A injections inhibit muscle growth^64^. In addition, it has been shown that the skeletal muscle tissue in rabbits did not fully recover six month post injection (3.5 UI/kg)^65^. When translating this finding into human years this would mean that the skeletal muscle tissue has not fully recovered 16 years after injection. However, there is no infallible mathematical formula to calculate the human age of a rabbit because its growth and physiological changes during it’s life are very different from the development seen in humans. And the existing age equivalence charts are usually based on observations of ages of rabbits from veterinarians. Nevertheless, the finding that high dose BoNT/A injections affect both bodyweight development, microstructure of the muscle and has an influence on the gait pattern of rats, raises a series of questions towards the rational of BoNT/A as a treatment against muscle contractures.

One major limitation of the present study is that the synchroton data are only analysed at one single timepoint (3 weeks post BoNT/A injection). This limits the ability to draw any major conclusions regarding the use of BoNT/A, since we cannot rule out that all the effects we observe here are fully reversible. Thus, long-term observations at several time-points are necessary in order to elucidate the recovery of the muscle tissue following BoNT/A injections. Furthermore, this study only reflects effects on the muscle tissue after one single injection. Future studies should investigate whether additional injections cause additive damage to the microstructure and the tissue. Another important issue in the present study is the fact that the injection volume is quite high (100 μl) compared to other studies were only 20 μl injection volume has been used^66^. The high injection volume has certainly produced some edema within the muscle tissue. However, the contralateral leg was injected with 100 μl saline saline as well and showed no signs of muscle damage 3 weeks after injection. However, it should be remembered that a high injection volume might increase the risk of systemic effects due to unintended spread of BoNT/A. One previous study has shown that high dose injections of botulinum toxin caused sporadic SNAP25 expression in distal muscles of rats indicating systemic spread but without evidence of transcytosis (dose: 20 µl of BoNT/A (3, 10, 30 U/kg)^67^. Unfortunately we were not able to investigate whether there was any systemic spread of BoNT/A in the present rats. However, it is possible that the high dose of BoNT/A injection and the high injection volume used in the present study might have caused systemic effects. These might be reflected by the reduced increase of body weight in the rats. On the other hand, the reduced increase of body weight in the BoNT/A injected rats may also be explained by reduced mobility of the rat due to the impaired muscle function caused by the local effect of the injection, but we cannot exclude that it may also be related to a systemic effect of the injection, although there were no visible signs of this.

The rats of present study did not lose much bodyweight; they rather showed a reduction of growth, unlike the animals used in the pilot study (Supplementary Material Figure 1). This difference in weight loss might be due to differences in age and baseline bodyweight. Unlike the rats that were used in the pilot study (Supplementary Material Figure 1) the rats from the present protocol were still growing, and this process might have counteracted the weight loss that was induced by the high dose BoNT/A injections.

## Conclusion

The present study leads to serious concerns regarding BoNT/A treatment because of the significant effect it has on the micro structure of the muscle tissue and tissue organization. Furthermore the results of the present study highlight that it is possible that intramuscular high dose BoNT/A injections might cause unwanted side effects such as muscle atrophy, and fatigue which causes an extensive remodeling process in the muscle tissue. The present findings indicate that high dose BoNT/A injections causes damage of the microstructure of the muscle tissue. Furthermore the present study shows that the physical capabilities are significantly reduced and the gait is significantly compromised 3 weeks after a high dose BoNT/A injection into the calf.

## Materials and Methods

### Animals

All experiments were conducted in accordance with the guidelines of EU Directive 2010/63/EU and were approved by the Danish Animal Experiments Inspectorate. 23 samples in the form of male Sprague Dawley rats (weight: 360 g) were used for the present experiment (n = 4 pilot gait analysis and clinical score (Baseline + 21 days post injection); n = 8 gait analysis and clinical score (21 days), n = 8 gene expression analysis and volume fraction (21 days post injection), n = 1 sham injection (Saline) gene expression normalization, and n = 2 control rats for body weight assessment. The rats were caged two by two (2 rats in each cage) in a 12/12 light dark cycle with access to water and food ad libitum. All recommended procedures for safe and proper handling, storage and preparation for experimental use, and disposal of Botulinum Toxin were complied. (https://www.cdc.gov/biosafety/publications/bmbl5/bmbl5_sect_viii_g.pdf). The entire medial gastrocnemius (including the injection site) of the muscle was dissected and harvested 21 days after BoNT/A injection and was used for gene expression analyses and volume fraction measurements. The tissue was harvested while the animals were anesthetized by 2% isoflurane. After the harvest the animals were euthanized using pentobarbital injections into the heart while the animals still were under anesthesia (2% isoflurane).

### Gait pattern analysis

The paws of both hind limbs of the rats were dipped in ink on a stamp pad. Then they were put down on a piece of graph paper and run through a plastic tunnel into a dark box. This procedure was then repeated three times. All paper strips were digitalized and analyzed using ImageJ (http://imagej.net/Welcome University of Wisconsin-Madison). The measurements of stride length, foot angle and foot length was done as indicated in Fig. 5.

### Tissue preparation

The rats were anesthetized by 2% isoflurane. The medial gastrocnemius was removed and dissected into smaller pieces with a scalpel (Swann-Morton, Mediq danmark A/S). The wet weight of the triceps surae was measured immediately after removal. One piece of the medial gastrocnemius was snap frozen in liquid nitrogen and stored at −80 °C for further PCR analysis. Another piece was fixed in Bouin’s fluid for 24 h and kept at 4 °C in 1.5 ml Eppendorf tubes. The tissue were then transferred into fresh tubes with 96% ETOH and remained at 4 °C until subsequently analysis.

The Clinical score assessment and gait analysis were conducted in four rats in a pilot study where baseline measurements and 21 days post measurements were obtained (Fig. 6). Subsequently eight rats were followed closely for 21 days and the clinical score and gait analysis was assessed every second day to monitor the acute effects over time after BoNT/A injection (Fig. 6).

### Injections

In order to test the dosage and injection volume a pilot study was conducted to test the optimal dosage (The optimal dose was defined as the smallest dose that would cause the desired effect of muscle atrophy, without causing any distress for the animals) (Results shown in Supplementary Material Figure 1). All animals were anesthetized with 2% isoflurane. Both hind limbs were shaved, and the skin was disinfected. Then a high dose of BoNT/A was injected (1 BoNT/A Unit = 10 picogram). The present high dose injection was 6 UI in total (6 UI = 60 pg in total) and (2 UI = 20 pg per 100 µl saline was injected per muscle head (Botulinum-toxin A (BoNT/A^®^ Allergan INC. Irvine CA)) of was injected into the triceps surae (medial gastrocnemius, the lateral gastrocnemius and soleus) muscle using a 0.5 ml syringe (Omnican^®^ 20 BRAUN, Germany), and 100 µl saline was injected per muscle head in the contralateral control leg. Each rat thereby received a total of 300 µl and 6UI of BoNT/A. Until termination of the experiment the welfare of the rats were routinely checked (e.g. for signs of dehydrations or distress). The rats were weighed every day following the injection in order to monitor weight loss. When any weight loss occurred the rats got 5 ml subcutaneous saline injections twice a day until the body weight was regained. The rats used in Clinical locomotion score assessment: The clinical evaluation system by (Malmsten 1983) was used to estimate the time course of improvement of motor performance in the hind limbs following BoNT/A injections^68^. The animals were tested at the following timepoints: 2 days pre injection and 2 days, 4 days, 7 days, 14 days and 21 days post injection. This is a system which scores the movement ability on a scale from 1 to 8 (1: no active movements of the limb, 2: few involuntary movements when handling the animal, 3: few uncontrolled gait movements with long breaks, 4: leg is used for locomotion without control, 5: leg is used for locomotion with little control, 6: leg is used for locomotion with increasing control, 7: Abnormal movements are only seen during close observation, 8: normal gait). The animals are observed while they walk voluntarily around in their cages for aprox 3 minutes, and the same observer rates the clinical score from 1 to 8.

### RNA extraction and real time-PCR analysis

Total RNA isolation: Total RNA was extracted from frozen muscle samples from n = 8 BoNT/A rats by using 1 ml of TRI Reagent (Molecular Research Centre, Cincinnati, OH) 5 steel beads (2.3 mm) and 1 silica bead (1.0 mm Silicon Carbide Beads (454 grams) BioSpec Products Inc.). Extracted RNA was precipitated from the aqueous phase with isopropanol and was washed with ethanol (75%), dried and suspended in 10 μl of nuclease-free water. The RNA concentration was determined using a RiboGreen RNA Quantitation kit 200–2000 Assays, Molecular Probes USA. RNA quality was determined on the basis of a RNA 6000 nano Chip assay kit, Agilent Technologies, Germany. The RNA samples were stored frozen at −20 °C until subsequent use in real-time RT-PCR procedures. To test the quality of the extracted RNA an electrophoresis in an agarose gel was made. The RNA quality was suggested to be satisfactory for further analysis.

### cDNA synthesis

150 ng RNA was reverse transcribed for each muscle sample in a total volume of 20 μl by using the Qiagen Omniscript RT Kit at 37 °C for 1 hour followed by 70 °C for 15 minutes. The resulting cDNA was diluted twenty times in dilution buffer (10 mM Tris EDTA buffer: Sigma Germany) + Salmon Testes DNA (1ng/μl; Sigma Germany), and samples were stored at −20 °C until used in the PCR reactions for specific mRNA analysis.

### Polymerase Chain Reaction

The Real-time PCR-method using Glyceraldehyde 3-phosphate dehydrogenase (GAPDH) and 60S acidic ribosomal protein P0 (RPLP0) as reference genes to study specific mRNA’s of interest was applied. However, since both reference genes were significantly affected of the BoNT/A injections, all data were normalized to the median values of the control samples of all animals. The primers were purchased from MWG Biotech. For each target cDNA the PCR reactions were carried out under identical conditions by using 5 μl diluted cDNA in a total volume of 25 μl QuantiTect SYBR Green PCR Mix (Qiagen) and 100 nM of each primer (Table 1). The amplification was monitored in real-time using a MX3005 P real-time PCR machine (Stratagene, CA). The threshold cycle (C_t_) values were related to a standard curve made with cloned PCR products to determine the relative difference between the unknown samples, accounting for the PCR efficiency. The specificity of the PCR reaction was confirmed by melting curve analysis after amplification. The real-time PCR conditions were as follows: to denaturate the DNA strands the reaction mix was heated above the melting temperature of DNA (95 °C) for 10 minutes, followed by 50 cycles each of 15 seconds at 95 °C, followed by the annealing step where optimal primer hybridization conditions were obtained by lowering the temperature to 58 °C for 30 seconds, and the extension step, where the reaction mix was heated to 63 °C for 90 seconds.

**Table 1:** PCR primers.

Gene	Sense	Anti sense	P-values
*RPLP0*	AGGGTCCTGGCTTTGTCTGTGG	AGCTGCAGGAGCAGCAGTGG	<0.001*
*GAPDH*	CCATTCTTCCACCTTTGATGCT	TGTTGCTGTAGCCATATTCATTGT	<0.001*
*Col1A1*	ATCAGCCCAAACCCCAAGGAGA	CGCAGGAAGGTCAGCTGGATAG	0.006*
*Col3A1*	TGATGGGATCCAATGAGGGAGA	GAGTCTCATGGCCTTGCGTGTTT	0.059
*DCN*	CACTCCAGGAGCTTCGACTCCAC	AGTGGGTTGCCGCCCAGTTC	0.131
*FMOD*	CCGTCAACACCAACCTGGAGAA	CGTGCAGAAACTGCTGATGGAGA	0.262
*FN1*	GGGCTTTGGCAGTGGTCATTT	CTCATCCGCTGGCCATTTTCTC	0.055
*GLUT4*	CTTCATCGTTGGCATGGGTTTC	CAAATGTCCGGCCTCTGGTTTC	0.594
*Itga7*	GCTGAGAAGAGAAACGTGAC	GTAGAGTGGGCAGCTGAATA	0.135
*Prelp*	CACCTGTACCTCAACAACAATA	GAAGTCATGGAAGGCCACTA	0.142
*IL6*	GACAAAGCCAGAGTCATTCAGAGCA	GAGCATTGGAAGTTGGGGTAGGA	<0.001*
*MMP2*	CTGGGTTTACCCCCTGATGTCC	AACCGGGGTCCATTTTCTTCTTT	0.017*
*TGFb1*	CCCCTGGAAAGGGCTCAACAC	TCCAACCCAGGTCCTTCCTAAAGTC	0.046*
*MHCIb*	ATTGCCGAGTCCCAGGTCAACA	GCTCCAGGTCTCAGGGCTTCAC	0.161
*MHCIIA*	GAAGAGCCGCGAGGTTCACAC	GGGACATGACCAAAGGCTTCACA	<0.001*
*MHCIIB*	GCCGAGTCCCAGGTCAACAAG	TGTGATTTCTTCTGTCACCTTTCAAC	<0.001*
*MHCIIX*	GCCGAGTCCCAGGTCAACAA	CTCATCTCTTTGGTCACTTTCCTGCT	0.042*

### Synchrotron radiation x-ray tomographic microscopy (SRXTM)

The SRXTM measurements were carried out at the TOMCAT (TOmographic Microscopy and Coherent rAdiology experimenTs) beamline at the Swiss Light Source^69^. The X-ray source is a superbending magnet radiation source located 25 m from the sample. A double crystal multilayer monochromator was placed 7 m downstream of the source to extract monochromatic X-ray photons at 25 keV. The detector system consisted of a 100 μm thick, Ce-doped LuAG (Lutetium Aluminum Garnet) scintillator which converted the transmitted X-rays into visible light, a high numerical-aperture microscope which gave a 20 fold magnification, and a pco.edge5.5 16-bit CMOS (Complementary metal–oxide–semiconductor) based camera to record the images. The resulting field-of-view (FoV) was 0.832 mm × 0.702 mm (width × height), and the effective pixel size was 325 nm. Muscle biopsies were placed in a 96% ETOH buffer in a small 0.2 ml Eppendorf tube and soldered to a holder with beeswax. The sample was placed 63 mm in front of the scintillator. Since the sample was larger than the FoV, local tomographic measurements were conducted by collecting 1501 projections of 600 ms exposure time over a 180 degree rotation. Flat-beam images (i.e., images taken with no sample) and dark images (i.e., images taken with no beam) were obtained in order to correct the projections. The total measuring time for all 21 samples took about 24 hours including sample mounting.

The 63 mm distance between sample and detector, combined with an effective pixel size of 325 nm, lead to refraction-induced intensity effects in the images. In order to account for this, refraction corrections were performed in using a local implementation at TOMCAT of a single image first order refraction correction algorithm. The algorithm used the Paganin approach with the assumption that the object consisted of a homogeneous soft tissue material and that the propagation distance was sufficiently short for the transport-of-intensity-equation to apply^30^. For tomographic reconstruction, a filtered back-projection based algorithm was applied.

### Image analysis

The background of the slices in the reconstructed tomogram suffered from a low-frequency bias-field. A correction was applied by first subtracting a constant plane and then subtracting a linear radial profile. The two correction functions were found by applying a least-squares fit to the mean of the tomogram stack slices.

For segmentation into a non-fibrillar and fibrillar phase, an alpha-level Markov random field (MRF) segmentation was applied to the tomograms as described by Pedersen *et al*.^70^. First, the data was modeled as a mixture of distribution functions by assigning a probability distribution to each phase. After assigning probability distributions, the spatial information of the data was incorporated into the segmentation process by modeling the data as an isotropic MRF^71^. The MRF smoothing parameter was set to 0.5. To find the optimal segmentation solution the multi-labeling problem was solved using graph cuts with alpha expansions as described in Boykov *et al*.^72^.

BoNT/A is expected to alter the microstructural tissue of the muscle and changes the fibrillary organization. This is evident from Fig. 1, where typical images are shown. Clearly, the ratio between the non-fibrillar and fibrillary structure is change significantly which indicates muscle atrophy and tissue damage. Furthermore, the anisotropy in the tissue structure is altered (3 weeks post BoNT/A injection, when compared to the contralateral control leg). In order to quantify these changes we have evaluated the tomograms.

Volume fraction (percent object volume). The tomograms visualize the muscle tissue around the injection site. From the segmented tomograms (3 weeks post BoNT/A injection, when compared to the contralateral control leg), volume fractions for the identified non-fibrillar and fibrillar phases were calculated as the percent object volume (POV) values. Image analysis as well as visualization of the tomograms was performed using custom made software implemented in MATLAB (Mathworks, Inc., Natick, MA).

### Anisotropy

The anisotropy analysis was performed 3 weeks post BoNT/A injection, and compared to the contralateral control leg, with a star length distribution (SLD) analysis using the Quant3D software as described elsewhere^73,74^. A volume of interest (VOI) from the non-fibrillar segmentation was used as an input to the SLD in Quant3D. For the orientation parameters, a uniform setting with 513 orientations, random rotations, and dense vectors were applied using 10,000 random points for calculating the SLD. The SLD analysis produces a number of anisotropic descriptors as described in^68^. As a measure of the orientation of the non-fibrillar tissue, the isotropy index was used. A value of one reflects a completely isotropic structure whilst zero reflects an anisotropic one. An assumption for the anisotropy analysis is that all the connected non-fibrillar tissue has been identified in the segmentation of the tomograms. Since the segmentation can have difficulties for samples with a very low non-fibrillar volume fraction, a lower threshold of 5% has been set. Two samples with a volume fraction below this threshold were excluded.

### Data availability

Due to the enormous amount of data, the datasets generated during and/or analysed during the current study are available from the corresponding author on reasonable request.

### Statistics

All data are presented as Mean ± SEM. Level of significance was set at 5% (p < 0.05).

### Clinical score

Changes in the clinical score from the second pre-test to the seventh post-test were investigated using an ANCOVA for repeated measures with time as an independent factor and the first pre-test value as a covariate. In case of significant main effect, a post hoc test was applied with Tukey correction for multiple comparisons. Gait analysis: Changes from the second pre-test to the fourth post-test in gait function measures (stride length, foot angle and foot length) were investigated using an ANCOVA for repeated measures with time and leg (BoNT/A leg and non BoNT/A leg) as independent factors and an interaction of time and leg. The first pre-test value was set as a covariate. In case of a significant main effect, a post hoc test was applied with Tukey correction for multiple comparisons. Volume fraction and anisotropy: Volume fraction and anisotropy was analyzed using a paired Students T-Test. Gene expression: Changes in gene expression between the BoNT/A leg and the control leg were investigated using a 2-Way ANOVA analysis with gene targets and leg (BoNT/A leg and control leg) as independent factors and an interaction of gene targets and leg. In case of a significant main effect, a post hoc test was applied with Tukey correction for multiple comparisons. All p-values from the post hoc tests are shown in Table 1. Changes in the development of body weight were investigated using a 2-Way ANOVA analysis with treatment and time as independent factors and an interaction of treatment and time. All PCR data are presented as the geo mean ± backtransformed SEM. Statistics software: Statistical calculations for gait analysis and the clinical score were performed in SAS Enterprise Guide (SAS Institute Inc. NC, USA 2015, version 7.11). The statistical calculations for changes in gene expressions and volume fraction were performed in Sigma Plot (Systat Software Inc. USA, version 12.5). Figures 1, 4 and 6 were generated in graphpad Prism 6.04 (GraphPad Software, Inc. CA, USA).

## Electronic supplementary material

Supplementary Information
