# Supplementary material for: Injection of high dose botulinum-toxin A leads to impaired skeletal muscle function and damage of the fibrilar and non-fibrilar structures
Source: Sci Rep. 2017 Nov 7;7:14746. doi: 10.1038/s41598-017-14997-3 (PMC5677119; doi:10.1038/s41598-017-14997-3)
Supplement: Supplementary file 1 — Supplementary Information [file 41598_2017_14997_MOESM1_ESM.pdf]

Supplementary material

Manuscript title:

Injection of high dose botulinum-toxin A leads to impaired skeletal muscle function and damage of the fibrilar and non-fibrilar structures.

Authors: Jessica Pingel*[1], Mikkel Schou Nielsen[2], Torsten Lauridsen[2], Kristian Rix[2], Martin Bech[3], Tine Alkjaer[1], Ida Torp Andersen[1], Jens Bo Nielsen[1], R. Feidenhansl[2†]

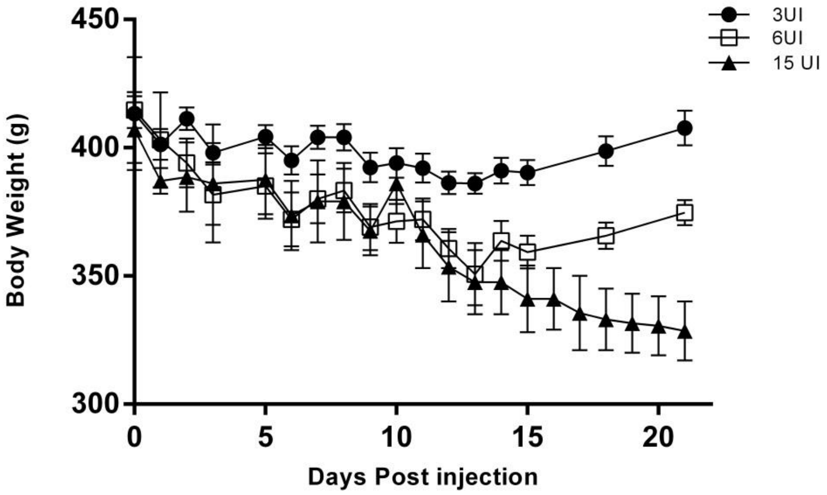

Figure Legends:

Figure I: The development of the body weight of the rats after BoNT/A injection. The closed squares represent injection of (3 UI=30 pg) (n=3), open squares represent injection of (6 UI= 60 pg) (n=3) and the closed triangles represent injection of (15 UI=150 pg) (n=3). All data are shown as delta Mean±SEM.
